# UTILIZATION OF ELDER CARE AMONG PEOPLE LIVING WITH DEMENTIA IN SWEDEN: A REGISTER-BASED STUDY

**DOI:** 10.1093/geroni/igad104.1547

**Published:** 2023-12-21

**Authors:** Atiqur sm-Rahman, Bettina Meinow, Lars-Christer Hydén, Susanne Kelfve

**Affiliations:** Linköping University, Norrköping, Ostergotlands Lan, Sweden; Stockholm Gerontology Research Center, Stockholm, Stockholms Lan, Sweden; Department of Culture and Society (IKOS), Linköping, Ostergotlands Lan, Sweden; Linköping University, Norrköping, Ostergotlands Lan, Sweden

## Abstract

The growing number of people living with dementia (PlwD) implies an increase in the demand for eldercare at different stages of the disease. This study aims to investigate the utilization of eldercare among people with and without dementia in Sweden during the last five years of life and what social-background factors influence the use of eldercare. Data were derived from four linked Swedish national registers comprising all decedents aged 70+ in Sweden as of November 2019 (n=6294). The primary outcome variable was the utilization of eldercare (no care, homecare, residential care). Following the study sample retrospective from death, data analysis was performed using multinomial and linear logistic regression models. Results showed that (1) nearly a quarter of all PlwD did not use any eldercare, primarily people who were newly diagnosed with dementia and living with partners; (2) three out of four PlwD used residential care in the last years of life; and (3) age, gender, and cohabitation status were important social-background factors determining utilization of eldercare for PlwD. This study provides unique insight that many Swedes with a dementia diagnosis do not receive any eldercare and that the utilization of eldercare increases with time since dementia diagnosis. We suggest more research to investigate why a substantial part of PlwD does not have any eldercare at all and what the policy implications of this might be.

